# *RHD* exon 5, 7 and 10 targeted non-invasive prenatal screening of fetal Rhesus-D (RhD) in selected RhD negative pregnant women in Ethiopia

**DOI:** 10.1371/journal.pone.0265583

**Published:** 2022-03-17

**Authors:** Birhanu Niguse, Mihertab Ermias, Solomon Berhanu, Lemma Abayneh, Bekele Chakiso, Riyaz Ahmad Rather

**Affiliations:** 1 Obsterics and Gynecology Unit, Nigist Eleni Mohammad Memorial Referral Hospital, Hossana, Ethiopia; 2 Department of Biotechnology, College of Natural and Computational Science, Wachemo University, Hosaena, Ethiopia; 3 Department of Biology, College of Natural and Computational Science, Wachemo University, Hosaena, Ethiopia; Universita degli Studi di Roma Tor Vergata, ITALY

## Abstract

**Background:**

A majority of non-invasive prenatal screening studies determining fetal RhD status have been tested on Caucasian and Asian populations, but limited or no studies have been conducted on the Ethiopian population. In the current study, we carried non-invasive prenatal screening of fetal *RHD* genotype in selected RhD negative Ethiopian pregnant women.

**Methods:**

Cell-free DNA was extracted from the plasma samples of 117 RhD pregnant women between 9 and 38 weeks of gestation. Fetal *RHD* genotypes were detected by targeting exons 5, 7 and 10 of the *RHD* gene by using real-time PCR assay. *RHD* genotypic results were confirmed by neonatal cord blood serology.

**Results:**

Fetal *RHD* genotyping was conclusive in all 117 subjects. *RHD* genotype was correctly predicted in 115 of 117 cases, thus the test yielded 98.3% accuracy (95%CI: 97.3–99.1%). Among 115 cases, 105 were genotyped as *RHD* positive and 12 were genotyped as *RHD* negative. The sensitivity and specificity of the test were 99.1% (95% CI: 94.8–99.9%) and 91.7% (95%CI: 61.5–99.7%) respectively. The negative and positive predictive values were 99.9% (95%CI: 99.2–99.9%) and 54.0% (95% CI: 15.2–88.4%) respectively. *SRY* genotyping results were in complete concordance with fetal sex.

**Conclusion:**

Multi exon targeted non-invasive prenatal screening test for fetal RhD determination exhibited high accuracy and sensitivity. A confirmatory study with a bigger size of study subjects is warranted before enabling clinical implementation.

## Introduction

Non-invasive prenatal screening (NIPS) is a method used to screen fetal anomalies or ascertain the genetic makeup of the fetus. The method utilizes the cell-free fetal DNA (cffDNA) or fetal originated cells circulating in the maternal plasma [1]. During pregnancy, fetal entities like cells or DNA is released into the maternal plasma by intact fetal cells, trophoblast burst or placenta shredding [2]. As the pregnancy advances, the fraction of fetal entities increases proportionally. These fractions, mostly cffDNA, are the key entities used in the NIPS setting. Reports indicate that cffDNA represents a small portion (3–6%) of total cell-free maternal plasma DNA [1]. Several analytical methods have predicted that cffDNA fragment size ranges from 150–200 bp [3]. To date, NIPS testing, centrally based on cffDNA has been used in the detection of trisomies, X-linked disorders, gender, pre-eclampsia, monogenic disorders and rhesus-D (RhD) type [4].

Prenatal determination of fetal RhD type is important in the management of pregnancies who are at the risk of alloimmunization. In adverse cases, alloimmunization can cause the lysis of fetal red blood cells that leads to haemolytic disease of the fetus and newborn (HDFN). Studies conducted on Caucasians for non-invasive determination of the fetal RhD type have provided substantial information on this non-invasive technique and thus this method has gained widespread recognition especially in the United Kingdom [5]. Other European countries like the Netherlands, Belgium, Denmark, Sweden, and France have introduced NIPS into routine clinical practice for the determination of fetal RhD status [6, 7]. The current NIPS technique used for the prediction of RhD blood type is based on- detecting the presence/absence of exon(s) and intron(s) sequences of the *RHD* or *RHCE* gene. The genomic structure indicates that the two genes are homologous and are located on chromosome 1, spanning each with ten exons and nine introns [8]. To date, multiple exons spanning the *RHD* were targeted to determine the fetal RhD status using maternal plasma. Antigen-D encoded by the *RHD* gene is the most significant rhesus blood group antigen. Owing to its high immunogenicity, it is the key cause of HDFN and transfusion reactions.

Despite experiencing key advances in this emerging technique in other parts of the globe, NIPS is yet to be tested or replicated on RhD negative expecting mothers in Ethiopia. Furthermore, despite having a 4–11% RhD negative blood group incidence rate in Ethiopia [9], no non-invasive fetal RhD genotyping studies have been conducted so far in Ethiopia. Hence, the current prospective study was undertaken to determine the fetal RhD blood type non-invasively in RhD negative pregnant women by targeting exon 5, 7, and 10 of the *RHD* gene.

## Methods

### Study setting

The study was conducted by employing 117 RhD negative pregnant women from the antenatal clinic of the Nigist Eleni Mohammad Memorial Referral Hospital, Hossana, Ethiopia, from June 2019 to March 2021. Study subjects that had evidence- of multiple pregnancies, intrauterine fetal death, fetal gross congenital malformations, history of blood transfusion in the last three months, and the ones that had RhD negative partners were excluded from the study. Written informed consent was obtained from all the study subjects, and ethical clearance (Memo no: WCU/EC/389/2019) was obtained from the University Ethics Review Board.

### Sample collection and plasma preparation

From each subject, 5 mL of peripheral blood was collected antecubically into EDTA Vacutainers and processed within 12 h. Briefly, samples were first centrifuged at 3000 ×*g* for 10 min to isolate the plasma. The isolated plasma was again re-centrifuged at 2700 ×*g* for 10 min to eliminate the free residual cells [10]. The plasma samples were stored at -80°C until further use. ABO and Rh typing of study subjects and cord blood samples was performed by standard agglutination procedure as per manufacturer’s protocol (Meril SA, Johannesburg, South Africa).

### DNA extraction and real-time PCR

Total DNA was isolated from 200 μL of plasma using QIAamp DNA Blood Mini Kit (Qiagen, Hilden, Germany). Final elution was done in 35 μL of elution buffer and the purity of the DNA was measured by spectrophotometer taking absorption at 260 and 280 nm. All the isolation procedures were carried out as per the protocol prescribed by the manufacturer.

The human *β-globin* and *SRY* gene were used as positive controls for whole DNA and fetal DNA extraction respectively. Real-time PCR (rt-PCR) using the TaqMan assay was carried on LightCycler 2.0 (Roche Diagnostics, Mannheim, Germany) using specific pre-tested primers and probes targeting towards exon 5, 7 and 10 of *RHD* gene besides *SRY* and *β-globin* gene [11]. All TaqMan probes were labelled with a reporter dye (FAM) at 5ˋend and with the quencher dye (TAMRA) at the 3ˋend (Table 1).

**Table 1:** Primer and probe characteristics used in the detection of fetal RhD status.

Primer	Sequence 5ˋ-3ˋ	GenBank	Product size (bp)	Ref.
Exon-7	CTCCATCATGGGCTACAA-F	BN000065	90	[11]
	CCGGCTCCGACGGTATC-R			
	AGCAGCACAATGTAGATGATCTCTCCA-P			
Exon-10	CCTCTCACTGTTGCCTGCATT-F		74	
	AGTGCCTGCGCGAACATT-R			
	TACGTGAGAAACGCTCATGACAGCAAAGTCT-P			
Exon-5	CGCCCTCTTCTTGTGGATG-F		85	
	GAACACGGCATTCTTCCTTTC-R			
	TCTGGCCAAGTTTCAACTCTGCTCTGCT-P			
SRY	TGGCGATTAAGTCAAATTCGC-F	L08063	137	
	CCCCCTAGTACCCTGACAATGTATT-R			
	AGCAGTAGAGCAGTCAGGGAGGCAGA-P			
β-globin	GTGCACCTGACTCCTGAGGAGA-F	U01317	102	
	CCTTGATACCAACCTGCCCAG-R			
	AAGGTGAACGTGGATGAAGTTGGTGG-P			

Each rt-PCR reaction was set up in a 20 μL reaction volume consisting of; 10 μL of TaqMan DNA Master Mix (Roche Diagnostics, Basel, Switzerland) and 5 μL of template DNA. Primer and probe volume was optimized in a manner so that they determine the minimum concentrations that gave the maximum reaction output. *RHD* exon 5, 7 and 10 primers were used at a concentration of 300 nM and *SRY* and *β-globin* were used at a concentration of 200 nM. All probes were used at a concentration of 200 nM and the final reaction volume was raised to 20 μL by adding nuclease-free water. All PCR amplification reactions were carried in 8 well PCR tubes in triplicates and the conditions were set as follows: Denaturation step of 10 min. at 95°C, followed by 50 cycles at 95°C for 15 seconds and at 60°C for 60 seconds. Samples tested as RhD negative were retested to confirm the absence of fetal DNA.

A sample was declared RhD positive if a fluorescent signal was detected in exon 5, 7 and 10 while as samples were declared RhD negative if no signal was obtained in any of the three exons. Results were declared inconclusive when only one or two signals among three tested exons were obtained. All reactions were carried in triplicates. Further, in the scenario of one or two positive detections out of three replicates in each tested exon, or in the event of discrepancies between the results of tested exons, the rt-PCR assay was scored inconclusive and was repeated with freshly isolated DNA from the same sample. In a situation of positive amplification in the *SRY* gene with no fluorescent signal in the *RHD* gene, the fetus was marked as an RhD negative male. If no amplification was detected in the targeted regions of the *RHD* and *SRY* gene, the fetus(es) were deemed as RhD negative females. Umbilical cord blood samples collected at birth were used to confirm the prenatal RhD genotyping results. To ensure quality, DNA isolated from known RhD positive and negative individuals was performed along with the tested samples. As per the local governing law, we didn’t disclose the gender outcome to the women till the baby was delivered.

### Statistical analysis

Statistical analysis was carried by using GraphPad Prism v8.4.1 (GraphPad Software, CA, USA). The specificity, sensitivity, positive and negative predictive values were determined by comparing the results of rt-PCR and RhD serological fetal or cord blood typing. All estimates were presented with 95% confidence intervals (CI) and a p-value less than 0.05 was considered significant.

## Results

**Fig 1:**
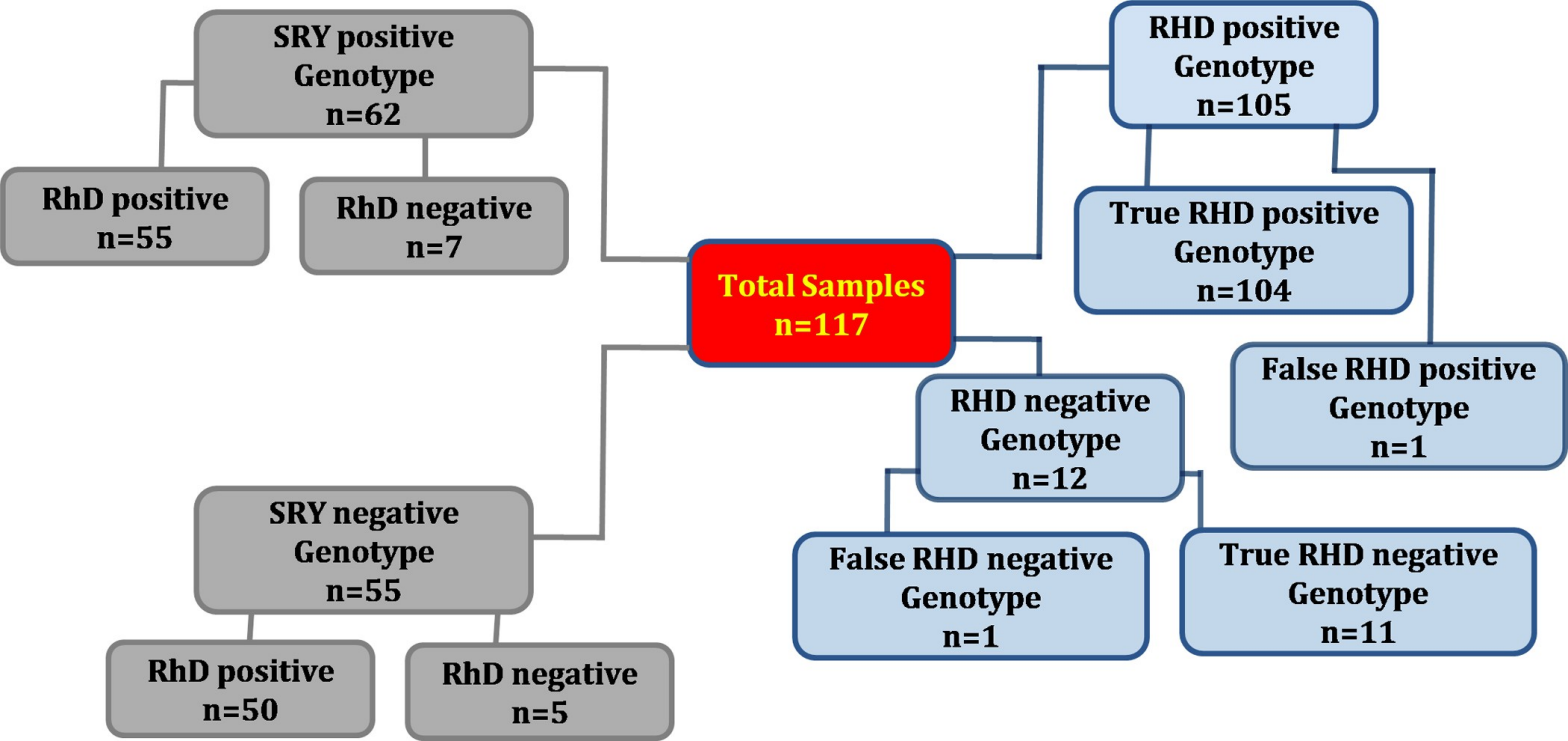
Summary of the results of non-invasive fetal *RHD* genotyping in maternal plasma.

NIPS test was conclusively evaluated in all the 117 plasma samples that were collected from pregnant women who tested RhD negative by routine serology. All recruited subjects were of African descent and live in the Hossana, Southwest of Ethiopia. The median gestational age at the time of sampling was 22±3 weeks (9–38 weeks). Twenty-one samples were gathered in the first trimester, whereas 67 and 29 were collected in the second and third trimesters of pregnancy respectively. Fetal *RHD* positive genotype was determined in 105 samples whereas 12 cases were genotyped as *RHD* negative (Fig 1). Among 105 positive predicted cases, 104 were correctly genotyped as *RHD* positive whereas 1 sample was identified as false positive. *RHD* negative genotype was correctly predicted in 11 cases with 1 false negative case reported. Neonatal RhD negative and positive blood type at the time of birth was 12 (10.2%) and 105 (89.8%) respectively. The test correctly predicted RhD phenotype in 115 of 117 cases, therefore the accuracy of the test was 98.3% (95%CI: 97.3–99.1%) (p<0.002). The sensitivity and specificity of the test were 99.1% (95% CI: 94.8–99.9%) and 91.7% (95%CI: 61.5–99.7%), respectively (Table 2). The negative and positive predictive values were 99.9% (95%CI: 99.2–99.9%) and 54.0% (95% CI: 15.2–88.4%), respectively.

**Table 2:** Correlation of neonatal blood serology with non-invasive prenatal screening test for *RHD* determination.

Neonatal blood serology			
NIPS test results	True Positive	False Positive	Positive Predictive Value
	104	1	54.0%
	False Negative	True Negative	Negative Predictive Value
	1	11	99.9%
	Sensitivity	Specificity	
	99.1%	91.7%	

NIPS; non-invasive prenatal screening.

Fetal sex outcome was known in all the pregnancies. *SRY* genotyping was in complete concordance with the newborn’s sex. Sixty-two (53.0%) fetuses were identified as *SRY* positive and fifty-five (47.0%) as *SRY* negative. Among sixty-two delivered male fetuses, fifty-five were RhD positive and seven as RhD negative. Of fifty-five delivered female fetuses, fifty were RhD positive and five were RhD negative.

## Discussion

The present study evaluated the fetal RhD status across all three trimesters of the pregnancy however, the majority of samples were drawn from the subjects recruited in the second and third trimesters. Limited or no visits to the antenatal care unit of the hospital during the early weeks of the pregnancy, confines the recruitment of the subjects, hence fewer subject recruitment was observed in the first trimester of the pregnancy. The data from the study area debate multiple underlying factors responsible for the limited antenatal care visits or loss to follow up in the early weeks of pregnancy [12]. Nevertheless, the diagnostic accuracy of NIPT is higher in the second and third trimesters of the pregnancy [13], however, we didn’t set this as a base parameter for the recruitment of our subjects. Previous studies have reported more than 98.0% diagnostic accuracy for RHD determination in samples taken at second or third trimesters of pregnancy whereas samples taken in the first trimester yielded lesser diagnostic accuracy [13–17]. Our diagnostic accuracy yield is in sync with earlier studies. Although the likelihood of getting false negative results in samples collected in the first trimester is higher, at the same time RhD fetal blood group determination in early gestation is very important in managing pregnancies that carry RhD positive fetuses.

It is recognized that the genetics of *RHD* and *RCHE* genes have a wider effect on RhD phenotype in an individual which further varies according to the ethnic origin of a person [18]. In particular, the RhD negative blood group manifests a different genetic variant which poses a big challenge in *RHD* genotyping. To circumvent this challenge it is advisable to use more than one target sequence for the assay determining fetal *RHD* genotype using maternal plasma samples. The current study targeted three different regions of the *RHD* to generate more sensitive and specific test results. We targeted a sequence within the 3՛ untranslated region of exon 10 of *RHD* which is a reliable predictor of RhD phenotype. Moreover, evaluation of exons 5 and 7 along with other exons of the *RHD* gene, have been demonstrated as good predictors of fetal RhD status in maternal plasma [5, 11, 16, 19]. Our three exon assay demonstrated an acceptable level of sensitivity (99.1%) and specificity (91.7%), which is in concordance with earlier studies. For instance, a recent study carried on the Croatian population showed 100% sensitivity and 95.08% specificity [19]. Another study conceded on 193 Italian subjects displayed 92.8% sensitivity and 94.1% specificity [20]. In the Netherlands, a nationwide screening program carried on 25789 RhD negative pregnant women demonstrated 99.94% and 97.74% sensitivity and specificity respectively [21]. Likewise, a screening program carried in Finland on 10814 women exhibited 99.9% sensitivity and 99.8% specificity [22]. In both these screening programs, the sample was collected from RhD negative pregnant women in the second trimester (24–26 weeks of gestation). Similarly, adequate literature is available from various studies across regions that suggest a wide range of reported sensitivity and specificity in the non-invasive prenatal determination of fetal RhD status [14].

The assay detected one false positive and negative case each. In the false negative case (NIPD-011), the sample was collected from the subject at 9.4 weeks of gestation. To make sure that the test result is a real false negative, we collected the second blood draw from NIPD-011 at 24.3 weeks of gestation and carried rt-PCR analysis again by targeting *SRY*, exon 5, 7 and 10. This time we detected a fluorescent signal for all the three tested exons along with *SRY*. On cord blood serology the sample was identified as RhD positive male fetus. Low concentration of cffDNA bearing RHD target sequences, in early weeks of gestation could be the possible reason for false negative RhD result or the rrt-PCR assay would not have been sensitive enough to detect the lower concentration of cffDNA. It is widely anticipated that plasma samples collected in the first trimester of pregnancy for non-invasive fetal RhD determination have a higher false negative detection rate as compared to samples collected in the second or third trimester of the pregnancy [23]. Further, cffDNA which may or may not bear fetal *RHD* target sequences enters into maternal plasma via feto-maternal trafficking and keep growing with advancing pregnancy [24]. With increasing gestational age, the concentration of cffDNA augments proportionally [10]. Owing to increasing cffDNA concentration, performing NIPS for determining fetal RhD status in second and third trimesters decreases the chances of prediction of false negative results.

In the falsely positive detected case (NIPD-67), the plasma sample was collected at 27.2 weeks of gestation. To make sure the result in this particular sample is truly “false positive”, we isolated the fresh DNA from the same sample and carried rt-PCR assay for the targeted exons, however, the repeated PCR analysis confirmed the false positive result. The occurrence of false positivity could be due to a variant *RHD* type. However, we could not confirm this hypothesis genotypically owing to limited resources. Previous studies have identified weak D, DEL or silent RHD variants as a determinant factor in producing false positive results [25, 26]. Variant RHD types like *RHD* pseudogene or *RHD*_*Ψ*_, are found in Africans and mostly occur due to inactive or silent *RHD* [27]. Weak D or variant *RHD* alleles are moderately present in African origin people as compared to Caucasian and white European race [28]. It is stressed in a previous study by Boggione et al. that the presence of *RHD* variants could lead to false positive results while performing fetal genotyping using plasma samples of RhD negative pregnant women [26]. Taking this incongruity into consideration a robust diagnostic algorithm based on molecular genotyping of paternal, maternal and fetal *RHD* type is advocated.

Approaches to confirm the presence of fetal DNA in the maternal plasma include amplification of the *SRY* sequences. However, this strategy is applicable only for pregnancies bearing a male fetus. No amplification of the *RHD* exon(s) and the *SRY*-specific sequences confirms either an RhD negative female fetus or a false negative result. The latter may be due to the low fetal DNA concentration in the maternal plasma. This creates anonymity within the results which cannot be determined until pregnancy outcome is known. Although our *SRY* genotyping had a 100% concordance with the newborn’s sex (Table 3), a universal internal positive control that detects fetal-specific sequences, is essential. One such marker *RASSF1A*, a tumor suppressor gene has been proposed as an internal positive control in recent times [29]. Chan et al., conducted a methylation-sensitive restriction enzyme digestion study on plasma samples collected from 71 first and third trimester, pregnant women. He observed a distinctive methylation pattern of the *RASSF1A* allele, wherein the restriction enzyme digested the maternal *RASSF1A* promoter sequence while leaving the promoter sequence of the fetal *RASSF1A* intact [29]. It was inferred that the promoter of the RASSF1A gene is hypomethylated in maternal blood cells and hypermethylated in fetal cells [29]. This distinctive methylation pattern differentiates between the fetal and maternal origin DNA.

**Table 3:** Correlation between *RHD* and *SRY* genotyping at different gestational weeks.

Gestation (Weeks)	Number	*RHD*+/*RHD*-	*SRY*+/*SRY*-	Concordance# (%)
9–13	21	19/2	12/9	100
14–26	67	60/7	32/35	100
27–38	29	26/3	18/11	100
Total	117	105/12 (89.7%/10.3%)	62/55 (52.9%/47.1%)	100

# concordant results confirmed by *SRY* genotyping with infant sex at the time of birth.

## Conclusion

We aimed this study to confirm the prediction of fetal RhD status using maternal plasma of RhD negative pregnant women in selected Ethiopian subjects. To our knowledge, this is the first attempt from the Horn of Africa to determine fetal RhD status non-invasively using the maternal circulatory plasma of RhD negative women. Despite having an experimental setup in a resource-limited setting, our assay results were 98.3% accurate. An acceptable sensitivity and specificity limits were achieved in this study. We suggest this assay be reproduced in different clinical settings of the country with larger and diverse patient sample sizes before implementing it into routine clinical practice. Although many countries adopted NIPS as a routine clinical screening test in expecting mothers, this report is the first attempt towards developing NIPS in the study area. It is to be noted that knowing fetal RhD status early in the pregnancy is very important in managing RhD pregnancies that are on verge of alloimmunization. Secondly, determining fetal RhD status early in the pregnancy will avert the need for unnecessary administration of immunoglobin prophylaxis in women who carry RhD negative fetuses. Therefore non-invasive prenatal determination of fetal *RHD* is very significant in managing Rh negative pregnancies. Hence all aspects of the test parameters should be purposefully investigated with a larger setup in the study area so that its clinical implementation becomes a vision to reality.
